# Supplementary material for: Fit for What Purpose? Exploring Bicultural Frameworks for the Architectural Design of Acute Mental Health Facilities
Source: Int J Environ Res Public Health. 2021 Feb 27;18(5):2343. doi: 10.3390/ijerph18052343 (PMC7956850; doi:10.3390/ijerph18052343)
Supplement: Supplementary file 1 [file ijerph-18-02343-s001.pdf]

Consent Form

Acute mental health ward design study
Mental Health Service User
Interview

I agree that:

1 I have read and I understand the Participant Information Sheet.

2 I speak English and understand that I will not have the option of using a translator.

3 I have been given sufficient time to consider whether or not to participate in this study.

4 I am satisfied with the answers I have been given about the study and I have a copy of this consent form and information sheet.

5 I understand that a staff member may be present during the interview.

6 I understand that the staff will be aware of my participation but that the information that I provide is confidential and will only be seen by the researchers in this study.

7 I understand that if I give the interviewer any reason to be concerned about my safety or that of someone else, that this information will be given to the lead clinician.

8 I understand that taking part in this study is voluntary (my choice) and that I can withdraw from the study at any time without penalty.

9 I consent to the research staff collecting and processing my information.

10 If I decide to withdraw from the study, I understand that the information collected about me up to the point when I withdraw will be removed from the data set and deleted.

11 I understand that no material which could identify me personally will be used in any reports on this study.

12 I know who to contact if I have any questions about the study in general.

13 I understand that my participation will involve a 30 minute interview with a researcher here in the ward.

14	I agree to having my bedroom in the ward photographed by the researcher.	Yes ☐	No ☐
15	I wish to receive a summary of the results from the study	Yes ☐	No ☐

If yes, email address:________________________________

Physical address:____________________________________

16	I agree to be contacted by the researcher by phone for any follow up questions.	Yes ☐	No ☐

If yes, phone number:_______________________________

Declaration by participant:
I hereby consent to take part in this study.

Participant's name: ____

Signature: ______________________ Date: ______________________

Declaration by member of research team:

I have given a verbal explanation of the research project to the participant, and have answered the participant's questions about it.

I believe that the participant understands the study and has given informed consent to participate.

Researcher's name: ____

Signature: ______________________ Date: ______________________

Participant Code:____

Demographics

Gender:
Age: _20-29 / 30-39 / 40- 49 / 50+_____

Ethnicity:

Which ethnic group do you belong to?
Please mark as many spaces as apply to you.

- ☐ New Zealand European
- ☐ Māori
- ☐ Samoan
- ☐ Cook Islands Maori
- ☐ Tongan
- ☐ Niuean
- ☐ Chinese
- ☐ Indian
- ☐ Other (Please state: eg Dutch, Japanese, Tokelauan)

____
____
____

Are you descended from a Māori (that is, did you have a Māori birth parent, grandparent or great-grandparent, etc)?

☐ Yes ☐ No ☐ Don't Know

Do you know the name(s) of your iwi (tribe or tribes)?

☐ Yes ☐ No

If yes, print the name and home area, rohe or region of your iwi below:

____
